# Development of silver-doped carbon dots sensor derived from lignin for dual-mode fluorometric and spectrophotometric determination of valsartan in a bulk powder and a commercial product

**DOI:** 10.1016/j.heliyon.2024.e40848

**Published:** 2024-11-29

**Authors:** Fatemah Aldakhil, Nawal A. Alarfaj, Salma A. Al-Tamimi, Maha F. El-Tohamy

**Affiliations:** Department of Chemistry, College of Science, King Saud University, P.O. Box 22452, Riyadh 11495, Saudi Arabia

**Keywords:** Valsartan, Carbon quantum dots, Green chemistry, Doping, Dual-mode sensor

## Abstract

Doping of carbon dots (CDs) with heteroatoms has garnered growing attention in recent years as a useful method of controlling their physicochemical properties. In this study, a new dual-mode sensor based on silver-doped CDs (AgCDs) derived from lignin was developed for fluorometric and spectrophotometric determination of valsartan (VAL). The analysis of AgCDs revealed a structure that closely resembled graphene oxide, with the successful doping of Ag. The mean particle size of AgCDs was 3.50 ± 0.89 nm and it exhibited a reasonable fluorescence quantum yield of 28.1 %. The emission at 612 nm of AgCDs is quenched by VAL after being excited at 275 nm due to a combination of dynamic and static quenching mechanisms. The enhancement in the absorbance of AgCDs upon the addition of the medication was measured at 275 nm. The most favorable circumstances for the dual-mode sensing were achieved with a pH of 8 and a volume of 0.10 mL of AgCDs. The measurements were conducted using fluorometry after 3 min at 10 °C, followed by spectrophotometry after 7 min at 20 °C. The fluorometric data indicated a linear response within the range of 2.0–50.0 μg/mL, while the spectrophotometric results showed a dynamic range of 5.0–100.0 μg/mL. The limits of detection (LODs) were 0.57 and 1.38 μg/mL for the fluorometric and spectrophotometric methods, respectively. The limits of quantification (LOQs) were 1.72 and 4.19 μg/mL for the fluorometric and spectrophotometric methods, respectively. The nano sensor efficiently assessed the presence of VAL in pharmaceutical tablets and produced a favorable outcome with the mean of recovery of 98.91 % and 99.76 % with relative standard deviation (RSD%) of 0.79 and 0.78 for the fluorometric and spectrophotometric methods, respectively.

## 1 Introduction

As a new “zero-dimensional” carbon nanomaterial, carbon dots (CDs) have gained significant attention due to its exceptional properties which include excellent biocompatibility, low toxicity, cost-effectiveness, great chemical stability, and extremely small size. As a result, CDs have been extensively studied for various appealing and innovative uses. The unique characteristics of CDs have sparked an increasing interest in their use as smart materials for many applications, such as bioimaging [1], fluorescence probing and analysis [2], photocatalysis [3], and photodynamic treatment [4]. Significant efforts have been dedicated to developing advanced fabrication procedures for specific features and applications. These efforts have been the subject of various research since 2006 [5].

Introducing heteroatoms into CDs is a highly successful method for controlling the physicochemical characteristics of CDs. In recent years, it has received considerable research focus because to its additional benefits compared to pure CDs. The interplay between the atomic orbitals of the heteroatoms and carbon atoms, along with the electron mobility induced by the heteroatoms, leads to modifications in the electronic structure, nanostructure, and chemical composition of heteroatom-doped CDs. Doping enhances the luminescence efficiency of CDs and introduces novel functionalities and active sites, significantly broadening their scope of applications [6,7].

Currently, there has been extensive research conducted on the study of CDs doped with non-metal elements such as N, B, S, P, and others [[8], [9], [10]]. Compared to the addition of non-metal elements, the incorporation of metals into the CDs matrix has shown finite development, which offers more opportunities for creating new types of doped CDs [11]. Introducing metals into CDs can alter the distribution of electron density and the energy gap of the CDs, resulting in noticeable modifications in their optical, electrical, and magnetic properties; metals possess a greater number of electrons, vacant orbitals, and a larger atomic radius compared to non-metallic atoms [12,13].

CDs can be produced in an eco-friendly and economical manner by using carbon-containing wastes as precursors, thus, transforming low-value waste materials into high-value nanomaterials [7]. Lignin, due to its aromatic conjugate structure, high carbon content, endogenous chemical modification and heteroatom doping, is an ideal carbon source for CDs [14]. Lignin can be directly converted into lignin-based CDs through hydrothermal treatment and can be improved by doping different heteroatoms such as Cl, N, S, metals, and others to enhance their optical properties [[15], [16], [17], [18], [19]]. The fluorescence QY of the lignin-based CDs described was evidently poor, and further investigation is required to explore the doping of heteroatoms into the lignin-based CDs. Sun et al. [14] devised a novel and simple technique for producing lignin-based CDs that exhibit a high fluorescent QY (43.9 %) and are sensitive to changes in pH. However, they used citric acid and ethylenediamine as precursors, along with lignin as a raw material.

Hypertension, a prevalent condition associated with aging, can impair the functioning of the heart and kidneys. The usage of antihypertensive medications alleviates the complications linked to hypertension. Valsartan (VAL), an angiotensin II receptor blocker (ARB), functions by inhibiting an endogenous compound that causes vasoconstriction in blood vessels. VAL reduces blood pressure and alleviates blood vessel constriction. Lowering blood pressure increases the supply of oxygen and blood to the heart. Several approaches, including spectroscopic, electrochemical, and chromatographic techniques, have been published for measuring the amount of VAL in biological fluids, pharmaceutical formulations, and bulk forms [[20], [21], [22], [23]].

One of the most important applications of CDs is fluorescent sensors, which are employed for detecting metal ions, anions, and molecules. Dual-mode sensing has emerged as a viable use of CDs in recent times [[24], [25], [26], [27], [28]]. Although many recent works rely on dual-mode detection using CDs [[29], [30], [31], [32]], the literature has not extensively explored the use of dual-mode CDs in drug sensing [33,34]. This study employed synthesized Ag-doped lignin-based CDs for drug sensing purposes and discovered that they exhibited exceptional selectivity and sensitivity towards VAL. A new, uncomplicated, and precise dual-mode sensor utilizing fluorometric and spectrophotometric methods is suggested for detecting VAL in both bulk powder and its commercial form.

## 2 Experimental

### 2.1 Materials and chemicals

High-grade chemicals and reagents were obtained from commercial providers and used without further purification. The following chemicals were acquired from Sigma Aldrich: sodium hydroxide (NaOH, 99.0 %), lignin, silver nitrate (AgNO_3_), hydrochloric acid (HCl, 37 %) and trisodium citrate dihydrate. Ethanol (99.9 %), sodium dihydrogen phosphate (99.5 %), and citric acid were acquired from BDH Laboratory supplies located in London, UK. Tabuk Pharmaceutical Co., based in Saudi Arabia, supplied the pure formulation of valsartan (98 %). The pharmaceutical product Diovan® 160 mg VAL/tablet, produced by Novartis Pharmaceutical Corporation in Switzerland, was obtained from local pharmacies in Riyadh, Saudi Arabia. Deionized water (DI water) was utilized for the preparation of the solutions and throughout the experiments. Polyethersulfone (PES) membrane filters, with a pore size of 0.22 μm, and dialysis tubing, with a cutoff molecular weight of 1000 Da (MD44), were purchased from Real Laboratory Supplies Store in China.

### 2.2 Instruments

Various spectroscopic and microscopic methods were used to confirm the synthesis of AgCDs. Photoluminescence spectroscopy, Fourier transform infrared (FTIR) spectroscopy, UV–Vis spectroscopy, energy dispersive X-ray analysis (EDX), and X-ray diffraction (XRD) were conducted using specific instruments: an RF-5301pc Shimazdu luminescence spectrometer equipped with a 150-W xenon arc lamp, a PerkinElmer FTIR spectrophotometer, an Ultrospec 2100 Biochrom spectrophotometer, a JSM-7610F JEOL EDX, and a Siemens D-5000 diffractometer, respectively. The produced AgCDs were examined using a transmission electron microscope (TEM-1400, JEOL Ltd.) and a scanning electron microscope (SEM-2100F, JEOL Ltd.) in order to gather microscopic data. The spectrophotometric measurements were conducted with an Ultrospec 2100-Biochrom spectrophotometer. The pH conditions of the samples analyzed were regulated using a pH meter (HANNA-Instruments HI 2211, Italy). A precise volume sampling was conducted using an Eppendorf electronic single-channel pipette (Repeater Stream) in all the tests.

### 2.3 Preparation of silver-doped CDs (AgCDs)

The AgCDs synthesis was conducted in a two-step process. Initially, silver nanoparticles (AgNPs) were synthesized using the methodology described by Yerragopu et al. [35]. In summary, a 100 mL solution of AgNO_3_ with a concentration of 1.5 mM was subjected to heating at a temperature of 90 °C for 5 min using a water bath. Subsequently, a volume of 12.5 mL of trisodium citrate solution (1.5 %) was slowly added to the mixture while continuously stirring. The reduction process initiates with a transition in color from translucent to a pale-yellow hue, signifying the creation of AgNPs. The nanoparticle solution was agitated at a temperature of 90 °C for 20 min and subjected to ultrasonic waves for 30 min.

The next step involves subjecting various quantities of AgNPs (2.00, 4.00, 8.00, 12.00 mL) to hydrothermal treatment. This is done by combining 1.50 g of lignin with 18.0 mL of a solution containing 2.0 mol/L NaOH. The resulting mixture is then subjected to sonication for 30 min. The hydrothermal treatment was conducted at a temperature of 220 °C for 16 h in an autoclave lined with Teflon. After the samples had cooled to the surrounding temperature, they were rendered acidic by adding HCl until the pH value reached 2. This resulted in the precipitation of any remaining unreacted lignin. Subsequently, the samples underwent sonication for 30 min, followed by centrifugation at a speed of 6000 rpm for 15 min. Subsequently, the samples underwent filtration using a 0.22-μm PES membrane. The liquid portions were moved to a dialysis bag with a molecular weight cutoff of 1000 Da. They were then subjected to dialysis against deionized water for 48 h to eliminate very small molecules and ions. Finally, the samples were stored at a temperature of 4 °C [36]. The solid products were acquired through freeze-drying for subsequent characterization.

### 2.4 Quantum yield measurements

The relative quantum yield (QY) of the AgCDs can be determined using the following formula:

$$\varphi_{X}=\varphi_{\text{std}}\times \frac{I_{X}}{{\;I}_{std}}\times \frac{A_{std}}{A_{X}}\times {\left(\frac{\eta_{X}}{\eta_{std}}\right)}^{2}$$

where the function φx represents the fluorescence QY, and x denotes the AgCDs. The chosen standard compound was quinine sulfate, which was dissolved in a solution of 0.1 M H_2_SO_4_ (φstd = 0.54). The refractive index, denoted by η, is equal to 1.33 for aqueous solution [10]. *A* refers to the absorption of light at the specific excitation wavelength of 350 nm, while *I* represents the integrated fluorescence intensity over the fluorescence emission spectrum. In order to reduce the impact of reabsorption, the absorbance at an excitation wavelength of 350 nm was modified to a value of 0.1 [27].

### 2.5 Dual-mode determination of VAL using AgCDs

**Preparation of stock solutions:** A stock solution containing AgCDs was made by mixing 1.00 g of AgCDs with 100.0 mL of deionized water. The resultant suspension was subjected to sonication for 10 min and subsequently stored at 4 °C. A stock solution (1000.0 μg/mL) of pure VAL was made by dissolving 0.10 g in 100.0 mL of ethanol.

**Detection procedure:** Aliquots of VAL (ranging from 2.0 to 100.0 μg/mL) were introduced into a set of 5-mL volumetric flasks. For the VAL solutions, 0.10 mL of the AgCDs was combined with approximately 1.00 mL of citrate-phosphate buffer at pH 8, along with DI water. The fluorescence spectra of the prepared solutions were measured 3 min after being cooled to 10 °C. The measurement was taken at a wavelength of 612 nm, with an excitation wavelength of 275 nm (ex:em slit = 5:5). After 7 min at 20 °C, a spectrophotometric method was used to quantify the absorbance at the maximum wavelength of 275 nm.

**Preparation of the samples:** The VAL pharmaceutical products' working solutions were made by pulverizing 10 Diovan® tablets, each containing 160 mg of VAL per tablet. 0.10 g of each powder were diluted with 100.0 mL of ethanol, subjected to ultrasonic waves for 10 min, and subsequently filtered. Additional dilution with DI water was performed to obtain analytical working samples. The drug's content was determined by employing a regression equation during the trial.

## 3 Results and discussion

### 3.1 Characterization of Ag-doped CDs

**Fig. 1:**
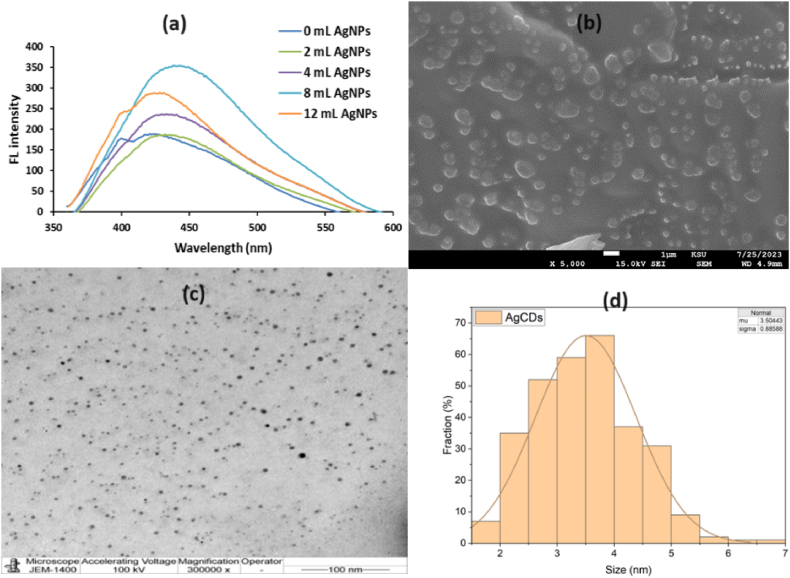
(a) fluorescence spectra of AgCDs with different amounts of AgNPs, (b) SEM image, (c) TEM image, and (d) Histogram of size distribution of AgCDs.

AgNPs were chosen for doping because of their exceptional conductivity, large surface area, catalytic activity, and biocompatibility [37]. Fig. 1a of AgCDs shows that fluorescence intensity increased when the added amount of AgNPs was increased from 0.00 to 8.00 mL, which was selected as the optimal AgNPs dosage per 1.50 g of lignin. The fluorescence intensity then decreased as the amount of AgNPs increased to 12.00 mL. The behavior of increasing and then decreasing fluorescence intensity upon increasing the dopant dose was also observed by Yang et al. [16] in the modification of lignin with ethylenediamine which may be due to the saturation of the active sites by the dopant.

The AgCDs were nearly spherical and uniformly distributed, as shown in Fig. 1b and c for SEM and TEM images, respectively. Although the particle sizes in the SEM (solid AgCDs) are larger than in the TEM image (soluble AgCDs), However, the SEM study could not provide any information about the shape and size match of the nanomaterials because of the aggregated particles [38]. Considering 300 particles by ImageJ software, the diameter distribution of AgCDs was in the range of 1.61–6.82 nm with an average diameter of 3.50 ± 0.89 nm (Fig. 1d). These results show that the AgCDs have a uniform quantum size distribution and excellent dispersion in water.

**Fig. 2:**
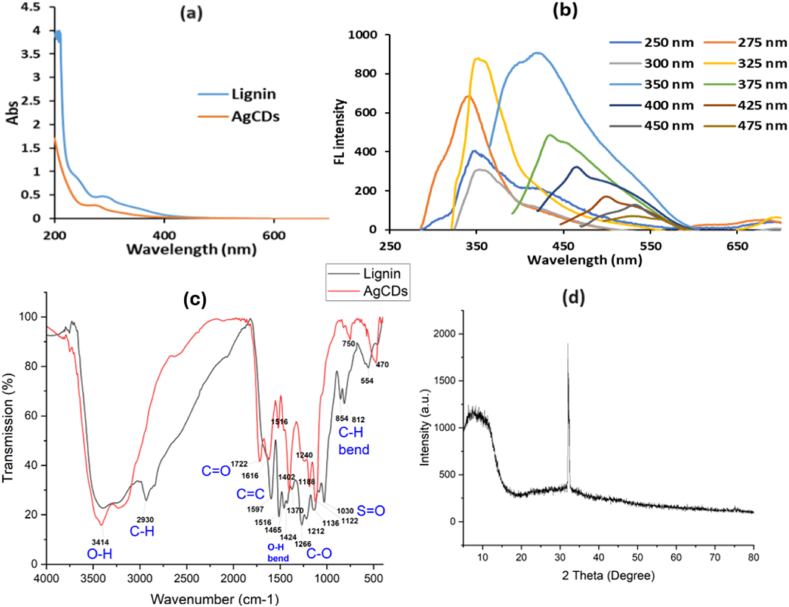
(a) UV–vis absorption spectra of lignin and AgCDs, (b) fluorescence spectra of AgCDs at different λex values, (c) FT-IR spectra of lignin and AgCDs, and (d) XRD spectrum of AgCDs.

The UV–vis spectroscopy was used to visualize the optical absorption behavior of the generated AgCDs. Fig. 2a clearly demonstrates the presence of a prominent peak for AgCDs at about 275 nm. This peak is attributed to the n→π∗ transition of the C=O band and the excited defect surface states caused by the heteroatoms [10,39]. AgCDs exhibited a blue shift to 275 nm in comparison to the UV–vis absorption peak of pure lignin (blue line) at 295 nm. The blue shift was likely caused by the aromatic ring substituents of the CDs, which resulted in an increase in steric hindrance. Consequently, there was a decrease in the degree of conjugation, making the n → π∗ electron transition more challenging. The absorption wavelength eventually decreased [14].

Fig. 2b displays the emission spectra of AgCDs under various excitation wavelengths. They demonstrate a phenomenon known as blue-shift, where there is a shift towards shorter wavelengths as the excitation wavelength increases from 250 to 275 nm. Additionally, they also display red-shift, which is a shift towards longer wavelengths as the excitation wavelength increases from 350 to 475 nm. The phenomenon of excitation-dependent fluorescence is often observed due to the size influence of various carbon cores and diverse surface states [40]. By comparing the quinine sulfate as a reference (with a QY of 54 % in a 0.1 mol/L H_2_SO_4_), the QY of AgCDs at 350 nm was determined to be 28.1 %. This value is greater than the synthesis from biomass waste mentioned in Ref. [41].

Fig. 2c shows the FTIR spectrum of AgCDs. The prominent peak at 3414 cm^−1^ was considered to be due to stretching vibrations of the O–H bond, and the peak at 2930 cm^−1^ was weak in AgCDs and was identiﬁed as C–H stretching. In addition, the strong peak at 1720 cm^−1^ was possibly attributed to the C=O bond. The absorption peaks at 1616 and 1516 cm^−1^ were attributed to C=C bonds. The detection of C=C bonds suggests a honeycomb-like structure of AgCDs, while the C–O–C group at 1402 cm^−1^ indicates that oxygen is doped into the planar structure [42]; this peak could also be attributed to O–H bending. The peaks of AgCDs at 1240, 1188, and 1122 cm^−1^ and the nearby peaks in lignin were assigned to C–O stretching of various groups (e.g., ethers, esters, and alcohols). Lignin has a strong peak at 1030 cm^−1^, possibly representing the S=O stretching of sulfoxide, but this peak becomes weak in AgCDs, which is due to the carbonization and doping processes. The C–H bend appears at 812-854 cm^−1^ in lignin and shifts to about 750 cm^−1^ in AgCDs. The previous findings suggest that the synthesized AgCDs have a variety of functional groups on their surface, resulting in a high level of hydrophilicity and stability in water. Additionally, they exhibit exceptional fluorescence performance, sensor specificity, and favorable biological compatibility [39,43].

The interlayer spacing was calculated according to the Bragg equation as [44]: 2 d Sin Ө = n λ where n is the order of Reflection (1), λ is the wavelength (0.15418 nm), and d is the interplanar spacing. The crystallographic pattern of AgCDs was analyzed from an X-ray diﬀractogram, as shown in Fig. 2d. A broad peak at around 2θ of 8.4° indicates the amorphous nature of AgCDs with graphene oxide-like structure (d-spacing 1.05 nm) [44]. Only the (1 1 1) basal plane of silver can be seen from the peak at about 2θ of 32°. The usual peaks ∼38 (2 0 0), ∼46 (1 2 0), ∼54 (2 0 2), and ∼77 (3 1 1) are not conspicuous in the diﬀractogram [45]. This may be due to the presence of CDs in a larger ratio than Ag, which may have resulted in a masking effect [38].

**Fig. 3:**
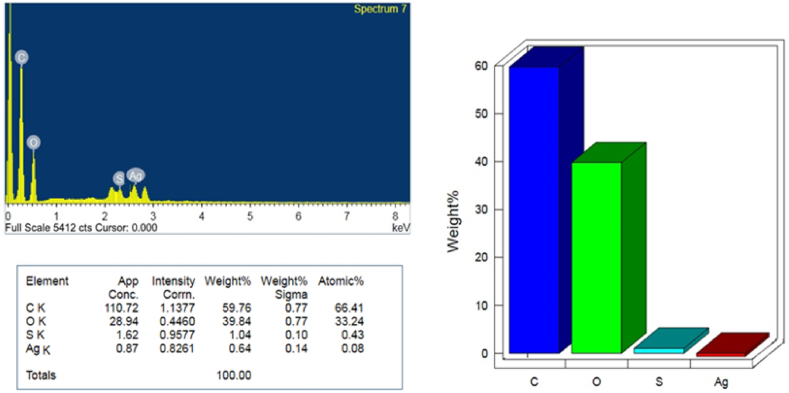
EDX analysis of the synthesized AgCDs.

An Energy Dispersive X-ray (EDX) examination was conducted to ascertain the chemical composition of the produced AgCDs. The EDX spectrum revealed the presence of Ag, C, O, and a minor quantity of S in the synthesized AgCDs. Fig. 3 displays the weight percentages and atomic percentages of the elements present in AgCDs. The absence of any other peaks corresponding to other elements in the produced AgCDs confirms their purity. The findings suggest a significant presence of oxygen in the sample, which aligns with the XRD results indicating a structure similar to graphene oxide and a large d-spacing. This can be attributed to the substantial number of oxygen-containing functional groups derived from the initial material (lignin).

**Fig. 4:**
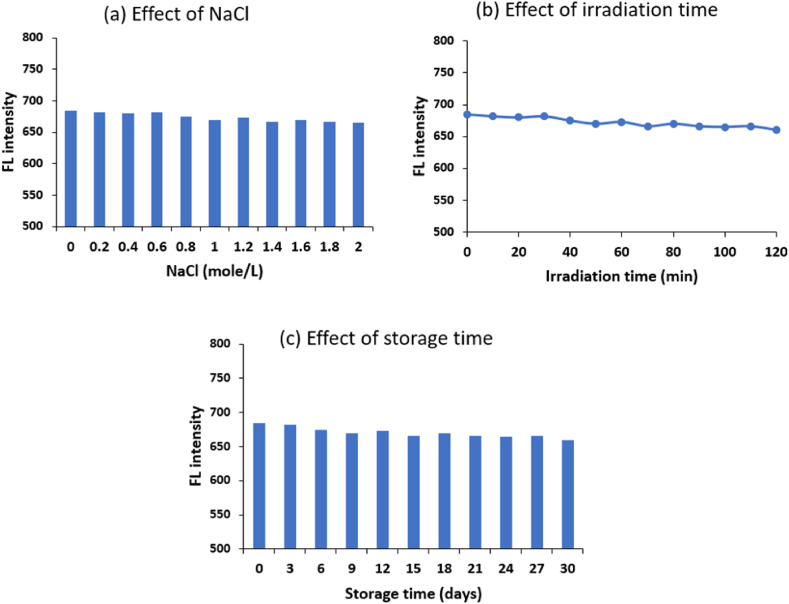
Factors affecting the stability of AgCDs: (a) effect of NaCl, (b) effect of irradiation time, and (c) effect of storage time.

The stability of AgCDs was assessed by examining their fluorescence properties in a saline solution, under a UV lamp, and after prolonged storage (Fig. 4). It was found that the fluorescence of the AgCDs remains almost unchanged when exposed to a high concentration NaCl solution (2.0 mol/L). After being exposed to a UV light for 120 min, there was no noticeable change in the fluorescence intensity of AgCDs, indicating a significant level of photostability. The AgCDs powder formed through lyophilization can be readily disseminated in deionized water without undergoing any agglomeration. Following a storage period of around 30 days at a temperature of 4 °C, the solution containing AgCDs maintained its visual integrity, showing no indications of separation or precipitation. Furthermore, the fluorescence intensity remains virtually unchanged. These observations indicate that AgCDs possess exceptional stability, which is beneficial for future applications.

### 3.2 Valsartan (VAL) determination

#### 3.2.1 Spectral characteristics

**Fig. 5:**
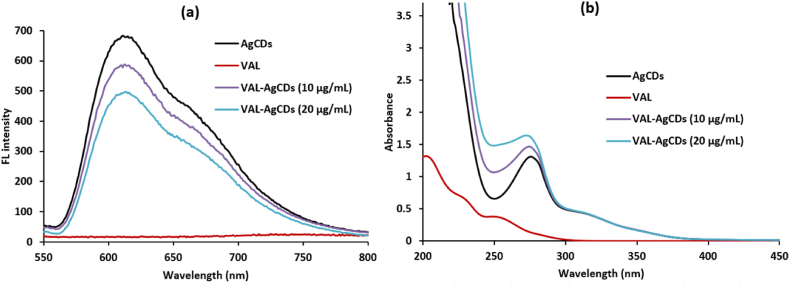
(a) fluorescence spectra and (b) absorption spectra of VAL solutions with different concentrations in the presence of AgCDs.

The addition of varied concentrations of VAL solution to the AgCDs resulted in varying degrees of alteration in the fluorescence intensity of the AgCDs. The magnitude of this change was influenced by the excitation and emission wavelengths. For instance, the alteration in VAL concentration did not exert a substantial influence on the disparity in fluorescence intensity (F_0_-F) when the excitation wavelength was set at 350 nm (F_0_ and F denote the fluorescence intensity of AgCDs without and with VAL, respectively). This could be due to the fact that the functional groups on the AgCDs, which are responsible for emitting fluorescence when excited at a wavelength of 350 nm, do not interact with the VAL compound [46]. Upon excitation of the AgCDs at a wavelength of 275 nm, the emission of the AgCDs at 612 nm is gradually quenched when VAL is added at various amounts, as shown in Fig. 5a. Therefore, the wavelengths of 275 nm and 612 nm were specifically chosen to excite and emit the VAL-AgCDs system, respectively.

On the other hand, the proposed spectrophotometric method was used to measure the amount of VAL, relying on the optical properties of the produced AgCDs. The UV–vis absorption spectra of the AgCDs with varying concentrations of VAL were acquired (Fig. 5b). The solutions of AgCDs, when VAL was added, exhibited a progressive rise in the absorbance peak at 275 nm, which was contingent upon the content of VAL. The suggested technique takes into account the difference in absorbance (A-A_0_), where A_0_ represents the absorbance intensity of the AgCDs in the absence of VAL, and A represents the absorbance intensity in the presence of VAL [27].

#### 3.2.2 Optimization of reaction conditions

**Fig. 6:**
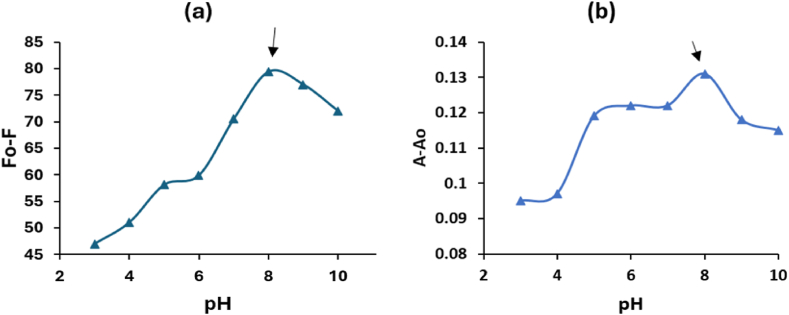
Effect of pH on the (a) F_0_-F and (b) A- A_0_ of 10 μg/mL VAL in the presence of AgCDs.

**Fig. 7:**
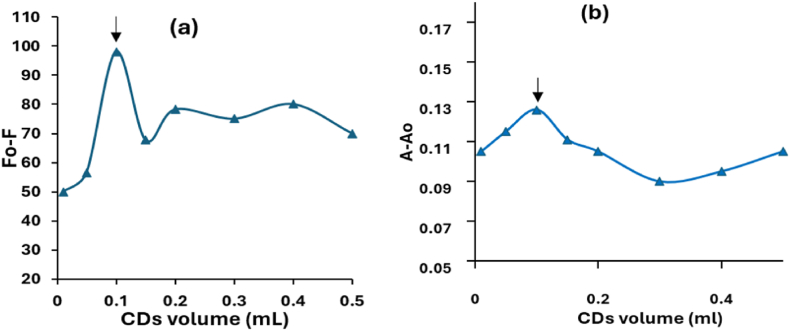
Effect of the AgCDs volume on the (a) F_0_-F and (b) A-A_0_ of 10 μg/mL VAL.

The impact of pH within the range of 3–10 was assessed while utilizing 0.10 mL of the AgCDs and a VAL solution with a concentration of 10.0 μg/mL. The highest F_0_-F and A- A_0_ values were observed when utilizing citrate-phosphate buffer with a pH of 8 for both procedures, as depicted in Fig. 6a and b, respectively. The optimal volume of the AgCDs, ranging from 0.01 to 0.50 mL, was determined by using a 10.0 μg/mL solution of VAL. The greatest values of F_0_-F and A- A_0_ were attained by introducing 0.10 mL of the AgCDs to the VAL solution, as depicted in Fig. 7a and b, respectively. Therefore, this volume was selected as the most optimal choice for carrying out further experimental research on this system.

**Fig. 8:**
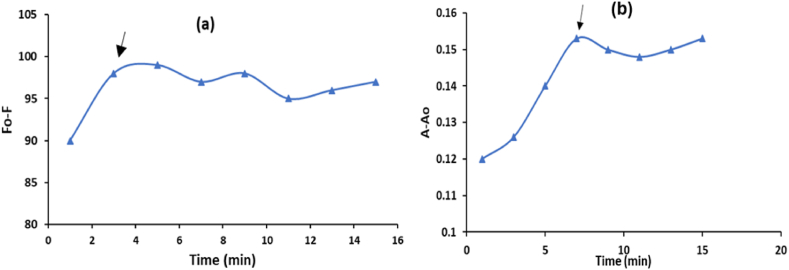
Impact of the response time on the (a) F_0_-F and (b) A-A_0_ of 10 μg/mL VAL in the presence of AgCDs.

**Fig. 9:**
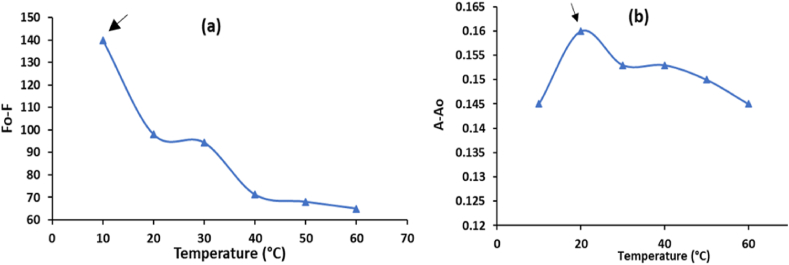
Effect of temperature on the (a) F_0_-F and (b) A-A_0_ of 10 μg/mL VAL in the presence of AgCDs.

The effect of the response time on the VAL samples and the added AgCDs was examined using a range of time intervals, from 1 to 15 min. The tested medication demonstrated quick responses within 3 and 7 min in the presence of AgCDs, as indicated by the results of fluorometric and spectrophotometric tests shown in Fig. 8a and b, respectively. A study was conducted to investigate the impact of temperature within the range of 10–60 °C on the fluorometric and spectrophotometric analysis of VAL. The experiment was conducted by either cooling the samples and the blank in an ice bath or by heating them in a water bath. The most effective quenching of AgCDs fluorescence by VAL was observed at a temperature of 10 °C, whereas the most significant increase in A-A_0_ happened at a temperature of 20 °C for the VAL-AgCDs system, as depicted in Fig. 9a and b, respectively. The ideal parameters for the suggested fluorometric and spectrophotometric methods are specified in Table 1.

**Table 1:** Optimized parameters for the measurement of VAL in the presence of AgCDs using the suggested dual-mode method.

Parameter	Studied range	Fluorometric method	Spectrophotometric method
λ_max_ (nm)	200–800	–	275
λex/em (nm)	250–800	275/612	–
ex: em slit	1.5–10 : 1.5–10	5 : 5	–
Buffer pH	3–10	8	8
CDs volume (mL)	0.01–0.50	0.10	0.10
Time (min)	1–15	3	7
Temperature (^o^C)	10–60	10	20

#### 3.2.3 Method validation

**Fig. 10:**
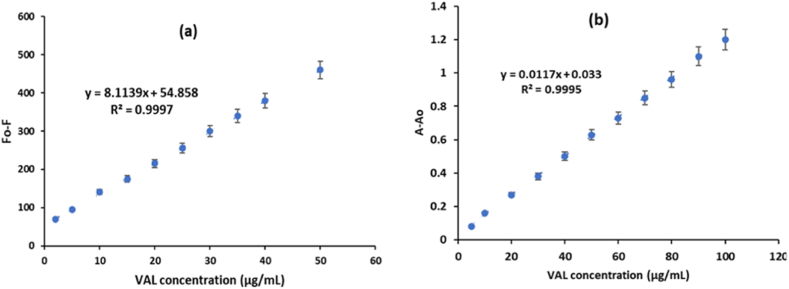
Calibration plots for the estimation of VAL in the presence of AgCDs using (a) fluorometric and (b) spectrophotometric methods.

A method validation was performed to verify the suitability as well as reliability of the suggested analytical approach for accurately and precisely detecting the substance being examined. A calibration plot was established to ascertain the concentration of VAL utilizing the recommended fluorometric technology. The fluorescence difference (F_0_-F) was plotted against the concentration of VAL, as shown in Fig. 10a. In addition, a spectrophotometric method was created by graphing the variation in absorbance (A-A_0_) against the concentration of VAL (Fig. 10b) [47]. The linearity of the graphs ranged from 2.0 to 50 μg mL-1 and from 5.0 to 100 μg mL-1 for VAL in the presence of AgCDs, as determined by fluorometric and spectrophotometric techniques, respectively. The statistical analysis of the obtained results revealed significant correlation coefficients (r) and small standard deviations for the intercept (Sa) and slope (Sb), indicating a high degree of linearity in the generated graphs (Table 2).

**Table 2:** Response data collected by determining VAL using the suggested dual-mode fluorometric and spectrophotometric methods in the presence of AgCDs.

Parameter	Fluorometric method	Spectrophotometric method
Concentration range (μg mL^−1^)	2.0–50	5.0–100
Slope	8.1	0.012
Intercept	55	0.033
Correlation coefficient (r)	0.9998	0.9998
Molar absorptivity (L. mol^−1^. cm^−1^)	–	5.1 × 10^3^
Sandell's sensitivity (μg cm^−2^/0.001 absorbance unit)	–	0.085
Standard Deviation of Intercept (S_a_)	1.4	0.0049
Standard Deviation of Slope (S_b_)	0.051	8.3 × 10^−5^
LODs (μg mL^−1^)	0.57	1.38
LOQs (μg mL^−1^)	1.72	4.19

The proposed procedure for detecting VAL in the presence of AgCDs proved to be highly sensitive by employing the following formulas to compute Sandell's sensitivity (SS) and molar absorptivity (*ε*): *ε* = $\frac{A}{C\times b}$ and SS = $\frac{0.001\times 1\;cm}{slope\;\left({cm}^{3}/\mu g\right)}$, where *A* represents the absorbance, *b* represents the path length in cm, and *C* represents the concentration in mol/L. Molar absorptivity measures the extent to which a chemical species absorbs light. Sandell's sensitivity is defined as the lowest concentration, expressed in μg/cm^3^, that results in an absorbance of 0.001 when light passes through a path length of 1.0 cm. The results obtained from the experiment, which demonstrated a high molar absorptivity and a low Sandell's sensitivity, indicate that the proposed approach exhibits a high level of sensitivity. The findings are briefly presented in Table 2.

The LODs and LOQs for determining the concentration of VAL in the presence of the AgCDs were calculated using the established fluorometric and spectrophotometric methods. The LOD was calculated as LOD = 3.3 Sa/slope. Similarly, the LOQ was found using the calculation LOQ = 10 Sa/slope. The obtained data is displayed in Table 2. The accuracy of the suggested fluorometric and spectrophotometric techniques was evaluated by conducting tests on ten and eleven authentic VAL samples, respectively. The results were evaluated by calculating the mean recovery percentage along with the standard deviation (mean ± SD). Both techniques exhibited outstanding detection ability with a high degree of accuracy, as evidenced in Table 3.

**Table 3:** The accuracy data acquired by determining VAL in bulk powder using the suggested fluorometric and spectrophotometric methods in the presence of AgCDs.

**Statistical analysis**	**Fluorometric method**			**Spectrophotometric method**		
	**Taken (μg/mL)**	**Found (μg/mL)**	**% Recovery**	**Taken (μg/mL)**	**Found (μg/mL)**	**% Recovery**
	2	1.940	97.00	5	4.960	99.20
	5	4.950	98.95	10	10.01	100.1
	10	10.20	102.0	20	20.24	101.2
	15	14.81	98.71	30	29.64	98.79
	20	19.74	98.68	40	39.89	99.72
	25	24.67	98.67	50	50.90	101.8
	30	30.21	100.7	60	59.53	99.22
	35	35.14	100.4	70	69.78	99.69
	40	40.07	100.2	80	79.18	98.97
	50	49.93	99.86	90	91.14	101.3
	–	–	–	100	99.68	99.68
**Mean ± SD**	99.52 ± 1.40			99.97 ± 1.02		
**n**	10			11		
**Variance**	1.96			1.04		
**%SE**^a^	0.44			0.31		
**%RSD**^b^	1.41			1.02		

a %SE = SD/√n.

b %RSD = (SD/Mean) × 100.

Both intraday and interday testing were used to assess the precision of the recommended strategy. The study investigated three separate dosages of the medication, with each dosage being tested three times (n = 3). The collected data was then evaluated using the relative standard deviation (RSD %) as a measure of variability. The average RSD % values for the determination of VAL for the intraday test in the presence of AgCDs were calculated to be 0.88 % and 0.92 % for fluorometric and spectrophotometric methods, respectively, as presented in Table 4. However, the interday test showed that the mean RSD% for the specified drug was 0.98 % and 1.10 % in the presence of AgCDs for fluorometric and spectrophotometric measurements, respectively. The obtained findings demonstrate a precision of the developed approach, with a deviation of less than 2 %.

**Table 4:** Intra- and interday precision of the VAL assay by the proposed fluorometric and spectrophotometric methods in the presence of AgCDs.

Precision test		Taken, μg/mL	% Recovery ±SD^a^	% RSD^b^	% Error^c^
**Fluorometric method**	Intraday precision	2	97.50 ± 1.00	1.03	0.59
		25	98.37 ± 0.76	0.77	0.44
		50	98.85 ± 0.83	0.84	0.48
	Interday precision	2	97.33 ± 1.53	1.57	0.91
		25	97.99 ± 1.14	1.17	0.67
		50	99.37 ± 0.21	0.21	0.12
**Spectrophotometric method**	Intraday precision	10	99.10 ± 0.69	0.70	0.40
		50	100.19 ± 1.13	1.13	0.65
		100	99.71 ± 0.92	0.92	0.53
	Interday precision	10	99.47 ± 1.33	1.34	0.77
		50	99.59 ± 0.71	0.72	0.41
		100	99.42 ± 1.25	1.25	0.72

a Mean of three determinations.

b %RSD= (SD/Mean) × 100.

c % Error = %RSD/√n.

By adjusting the procedure parameters slightly, the robustness of the fluorometric and spectrophotometric detection methods for VAL determination was investigated. By changing the AgCDs' volume (±0.01 mL) and pH (±1.0), the robustness of the methods was evaluated. These modifications had no effect on the difference in fluorescence (F_0_-F) or the difference in absorbance (A-A_0_). The percent recoveries for the fluorometric and spectrophotometric studies in the presence of AgCDs were found to be 98.51 ± 1.61 % and 99.81 ± 1.06 %, respectively.

By evaluating the same samples in several settings ‒ different laboratories, different operators, and different equipment ‒ the ruggedness of the fluorometric and spectrophotometric detection methods for the determination of VAL was examined. These adjustments had no effect on the fluorescence difference (F_0_-F) or the absorbance difference (A-A_0_). For both fluorometric and spectrophotometric methods, the obtained percent recoveries in the presence of AgCDs were 99.47 ± 1.01 % and 99.05 ± 0.99 %, respectively.

**Fig. 11:**
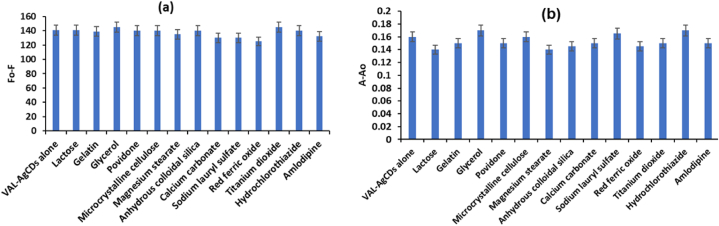
Selectivity of the VAL-AgCDs system in the presence of different species for (a) fluorometric and (b) spectrophotometric methods at VAL concentration of 10 μg/mL.

In order to investigate the response of AgCDs to different species, which include VAL, lactose, gelatin, glycerol, microcrystalline cellulose, povidone, anhydrous colloidal silica, magnesium stearate, calcium carbonate, sodium lauryl sulfate, titanium dioxide, red ferric oxide, hydrochlorothiazide, and amlodipine, we configured AgCDs into 14 parts of water solution of the same concentration, adding different species of the same concentration to these 14 parts of water solution. We found by visual observation that the above species (except VAL) did not significantly change the fluorescence and absorbance of AgCDs, but only VAL can selectively quench the fluorescence and enhance the absorbance of AgCDs. Therefore, the proposed fluorometric and spectrophotometric methods for determining VAL were assessed for their selectivity in the presence of different coadditives and potential interfering species found in pharmaceutical preparations [48]. Fig. 11a displays the alterations in the fluorescence intensity when the coadditive species are present in the VAL-AgCDs system. Fig. 11b illustrates the alterations in the absorbance difference (A-A_0_) when the coadditive species are present in the VAL-AgCDs system. The results indicate minimal impact on the fluorescence and absorbance of the system. Therefore, the suggested dual-mode fluorometric and spectrophotometric method for quantifying VAL can be regarded as a selective approach.

The quantity of the tested constituent in the pharmaceutical formulation (Diovan® 160 mg VAL/tablet) was determined using the suggested analytical technique, and the results are presented in Table 5. The fluorometric method produced a calculated value of 98.91 ± 0.78, whereas the spectrophotometric method produced a calculated value of 99.76 ± 0.77 when AgCDs were present. The data underwent statistical analysis using Student's t-test and the variance ratio F-test. The obtained results were also compared to the findings of a previously published technique (UV method) [49].

**Table 5:** Application of the proposed and comparison methods for the determination of VAL in its pharmaceutical formulation (Diovan® 160 mg VAL/tablet) in the presence of AgCDs.

**Statistical analysis**	**Fluorometric method**		**Spectrophotometric method**	
	**Taken (μg mL**^**−**^**^1^)**	**% Recovery**	**Taken (μg mL**^**−**^**^1^)**	% **Recovery**
	2	97.50	5	99.20
	10	99.20	10	100.2
	20	98.75	30	98.50
	30	99.33	50	100.5
	40	98.88	70	100.3
	50	99.80	100	99.79
**Mean ± SD**	98.91 **±** 0.78		99.76 ± 0.77	
**n**	6		6	
**Variance**	0.61		0.60	
**%SE**^a^	0.32		0.32	
**%RSD**^b^	0.79		0.78	
***t*-test**	2.16 (2.228)^c^		0.18 (2.228)^c^	
**F test**	3.65 (5.05)^c^		3.56 (5.05)^c^	
**Reported method (**UV method) [49]	99.69 **±** 0.41 (n = 6)			

a % SE = SD/√n.

b % RSD **=** (SD/Mean) × 100.

c The figures between parentheses are the tabulated t- and F values at p < 0.05.

The current approach for determining VAL, which combines fluorometric and spectrophotometric techniques, is either superior or comparable to previously documented methods (as shown in Table 6). These methods include spectrophotometric [21], electrochemical [23], fluorescence [50], and chromatographic [51] techniques. The created nano sensor is noteworthy for its eco-friendliness, high selectivity, and ease of production. Furthermore, it relies on uncomplicated and easily accessible tools, harmless substances, and gentle synthetic environments. In addition, it demonstrates exceptional accuracy and precision and does not necessitate considerable technical proficiency, unlike alternative chromatographic or electrochemical techniques.

**Table 6:** A comparative study between the results obtained from the determination of VAL by the proposed dual-mode fluorometric and spectrophotometric method and previously reported analytical techniques.

Analytical Technique	Reagent	Linearity	LOD	Reference
Spectrophotometry	VAL and nebivolol dissolved in methanol	4–80 μg mL^−1^	1.183 μg mL^−1^	[21]
Electrochemical	VAL and amlodipine, boron-doped diamond electrode	19.8–280 μM	0.193 μM	[23]
Fluorescence	VAL, McIlvaine buffer pH3, in addition to nanoparticles	0.03–0.27 μg mL^−1^	0.004 μg mL^−1^	[50]
Chromatography	VAL, propranolol, acetonitrile, methanol, and disodium hydrogen phosphate (pH 3.5)	4–32 μg mL^−1^	0.45 μg mL^−1^	[51]
Proposed method	VAL, AgCDs and citrate-phosphate buffer (pH 8)	2–50 μg/mL	0.57 μg/mL	Fluorometric method
		5–100 μg/mL	1.38 μg/mL	Spectrophotometric method

#### 3.2.4 Detection mechanism

The interaction between a quencher molecule and a fluorophore is the primary quenching mechanism in sensing. This phenomenon arises as a result of either dynamic or static collisions between the quencher and the fluorophore (CDs). Static quenching is the result of the formation of a nonfluorescent ground-state complex due to the interaction between CDs and the quencher. Upon light absorption, the complex promptly reverts back to its ground state without emitting a photon. Dynamic quenching occurs when the excited state of CDs transitions back to the ground state due to collisions between the quencher and CDs, through either energy transfer or charge transfer mechanisms [52].

**Fig. 12:**
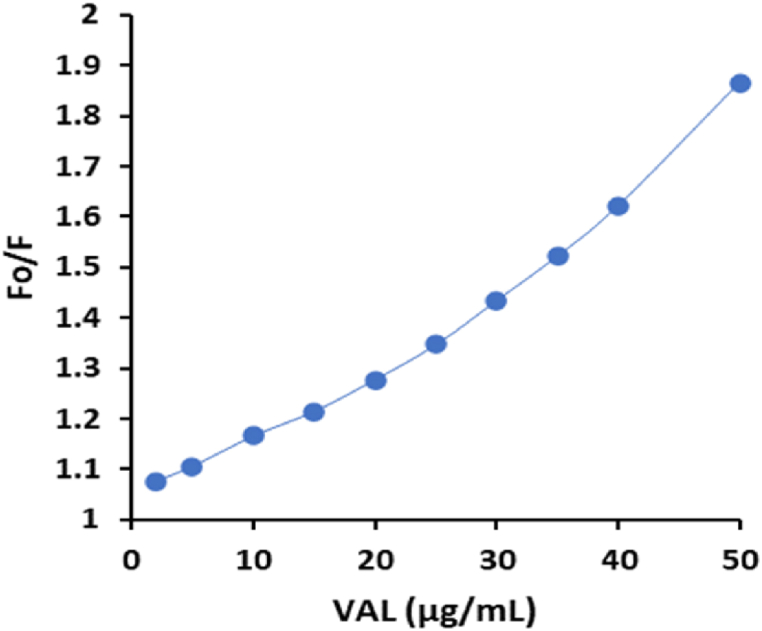
Nonlinear Stern–Volmer plots for the VAL-AgCDs system.

In order to understand the mechanism of fluorescence quenching of VAL-AgCDs system, the fluorescence quenching data were examined using the Stern-Volmer (S-V) equation, which is presented below: F_0_/F = 1 + Ksv [Q] where F_0_ and F are the fluorescence intensities of the AgCDs in the absence and presence of VAL, respectively. The concentration of the quencher (VAL) is denoted as [Q], and the S−V quenching constant of the AgCDs is represented as Ksv. The S−V plots depicted in Fig. 12 for the VAL-AgCDs system illustrate a steady drop in fluorescence intensities of the AgCDs (resulting in a higher F_0_/F) upon the addition of 2–50 μg/mL VAL. The results demonstrate that VAL may efficiently suppress the fluorescence of the AgCDs. Nevertheless, the S−V plots of the VAL-AgCDs system do not exhibit a typical linear correlation between the F_0_/F ratio and the concentration of quencher. Consequently, it was hypothesized that both static and dynamic quenching processes contribute to the quenching process in this system [53]. The equation that describes the polynomial curve is the S-V equation, which is given by:

$$F_{0}/F=\left(1+K_{\text{SV}}\;\left[Q\right]\right)\;\left(1+K_{S}\;\left[Q\right]\right)=1+\left(K_{\text{SV}}+K_{S}\right)\;\left[Q\right]+K_{\text{SV}}\;K_{S}\;{\left[Q\right]}^{2}$$

In this equation, Ksv represents the dynamic quenching constant and Ks represents the static quenching constant [54,55].

## 4 Conclusions

This paper describes the synthesis of a dual fluorometric/spectrophotometric AgCDs sensor with excellent water solubility and strong emission. The synthesis process consists of a two-step procedure that involves the use of AgNPs and lignin dissolved in NaOH. This nano sensor, which has a high level of selectivity and a broad linear range, was effectively utilized to determine the presence of VAL in both a bulk powder and a commercially available product using fluorometric and spectrophotometric methods. The emission of AgCDs at 612 nm is quenched by VAL after being excited at 275 nm due to a combination of dynamic and static quenching mechanisms. The enhancement in the absorbance of AgCDs upon the addition of VAL was measured at 275 nm. The most favorable conditions for dual-mode sensing were achieved with a pH of 8 and a volume of 0.10 mL of AgCDs. The measurements were conducted using fluorometry after 3 min at 10 °C, followed by spectrophotometry after 7 min at 20 °C. This discovery is important for furthering the development and application of a dual-mode nano sensor that utilizes CDs in the field of drug detection.

## CRediT authorship contribution statement

**Fatemah Aldakhil:** Methodology, Investigation, Data curation. **Nawal A. Alarfaj:** Supervision, Funding acquisition, Conceptualization. **Salma A. Al-Tamimi:** Supervision, Resources, Formal analysis. **Maha F. El-Tohamy:** Writing – review & editing, Visualization.

## Data availability

The data substantiating the results of this investigation have been included in the publication.

## Funding information

This research was funded by Researchers Supporting Project number (RSP2024R272), 10.13039/501100002383King Saud University, located in Riyadh, Saudi Arabia.

## Declaration of competing interest

The authors declare that they have no known competing financial interests or personal relationships that could have appeared to influence the work reported in this paper.
